# Antimicrobial resistance in methicillin-resistant staphylococcus aureus

**DOI:** 10.1016/j.sjbs.2023.103604

**Published:** 2023-02-28

**Authors:** Bandar Ali Alghamdi, Intisar Al-Johani, Jawhra M. Al-Shamrani, Hussein Musamed Alshamrani, Bandar G. Al-Otaibi, Kholod Almazmomi, Nik Yusnoraini Yusof

**Affiliations:** a Department of Cardiac Surgery, King Fahad Armed Forces Hospital, Jeddah, Saudi Arabia; b Department of Biotechnology, Taif University, Taif City, Saudi Arabia; c Directorate of Health Affairs in Qunfudah Center (Namerah Primary Health care) Pharmacy Department, Saudi Arabia; d Albandr Clinic, Taif, Saudi Arabia; e Institute for Research in Molecular Medicine (INFORMM), Health Campus, Universiti Sains Malaysia, Kubang Kerian 16150, Kelantan, Malaysia

**Keywords:** MRSA, Antibiotics, Antimicrobial resistant bacteria, Methicillin resistance mechanism of MRSA Methicillin resistance mechanism of MRSA

## Abstract

In the medical community, antibiotics are revered as a miracle because they stop diseases brought on by pathogenic bacteria. Antibiotics have become the cornerstone of contemporary medical advancements ever since penicillin was discovered. Antibiotic resistance developed among germs quickly, placing a strain in the medical field. Methicillin-resistant *Staphylococcus aureus* (MRSA), Since 1961, has emerged as the major general antimicrobial resistant bacteria (AMR) worldwide. MRSA can easily transmit across the hospital system and has mostly gained resistance to medications called beta-lactamases. This enzyme destroys the cell wall of beta-lactam antibiotics resulting in resistance against that respective antibiotic. Daptomycin, linezolid and vancomycin were previously used to treat MRSA infections. However, due to mutations and Single nucleotide polymorphisms (SNPs) in Open reading frames (ORFs) and SCCmec machinery of respective antibody, MRSA developed resistance against those antibiotics. The MRSA strains (USA300, CC398, CC130 etc.), when their pan-genomes were analyzed were found the genes involved in invoking resistance against the antibiotics as well as the epidemiology of that respective strain. PENC (penicillin plus potassium clavulanate) is the new antibiotic showing potential in treatment of MRSA though it is itself resistant against penicillin alone. In this review, our main focus is on mechanism of development of AMR in MRSA, how different ORFs are involved in evoking resistance in MRSA and what is the core-genome of different antimicrobial resistant MRSA.

## Graphical abstract

**Figure:**
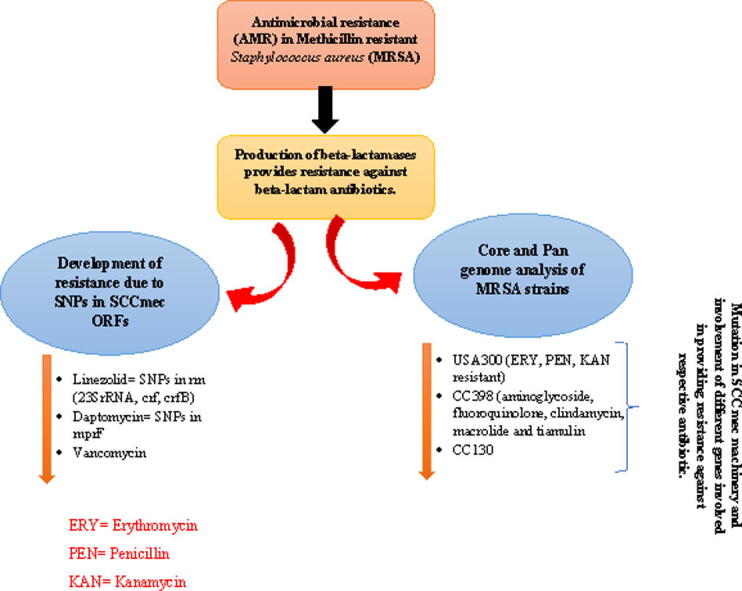

## 1 Introduction

One of the greatest medical advancements in the treatment of infectious disorders brought on by infectious bacteria is the antibiotic development. Before discovery of antibiotics, the mortality and lethality brought on by infectious bacteria was significant (Hutchings et al., 2019). Alexander Fleming accidentally discovered penicillin again in 1928. The investigation of more antibiotic kinds, including fluoroquinolones, aminoglycosides, lipopeptides, sulphonamides, and many others, is made possible by this rediscovery (Hutchings et al., 2019, Nicolaou and Rigol, 2018). The ability of antibiotics to help prevent infection in chemotherapy patients and other surgical wounds also makes it possible for contemporary medical technologies to exist.

Despite the fact that antibiotics have a substantial advantage in the treatment of diseases brought on by infectious bacteria, Fleming’s cautions against the risk of unrestrained use of antibiotics, which can lead to the development of resistance. *Escherichia coli* first showed signs of antibiotic resistance (AR) to penicillin in 1940, indicating that the warning was accurate (Abraham and Chain, 1988). Since more germs became resistant to different types of antibiotics, it has been a major concern to the medical field up until this point. According to estimates, 10 million people could die each year from AR by the year 2050 (Dixit et al., 2019, Lv et al., 2021).

According to a recent in-depth analysis published in The Lancet, 204 nations are expected to account for AR-associated deaths (4.95 million) and AR-attributed deaths (1.27 million) in 2019. Western Sub-Saharan Africa has the highest rate of AR-related deaths, with an anticipated 114.8 AR-associated death rate/1lac and 27.3 AR-attributed mortality rate/1lac people. Australia had the lowest fatality rate, with only 28 AR associated mortality per 100,000 people and 6.5 AR-attributed fatalities per 100,000 100,000 people. The six pathogenic germs that will cause the most fatalities in 2019 are listed in the same report (Murray et al., 2022). *Pseudomonas aeruginosa, Streptococcus pneumoniae, E. coli, Acinetobacter baumannii, Staphylococcus aureus, and Klebsiella pneumoniae* caused the most AR related deaths (3.57 million), followed by 929,000 AR attributed fatalities.

*Staphylococcus aureus*, as a prevalent member of body's microbiota, is a Gram + ve bacteria having a spherical morphology. Although this bacteria lives on humans commensally, it can nonetheless be voracious as it can lead to food poisoning and skin infections. Antibiotic-resistant (mainly methicillin-resistant) variant of *S. aureus* known as MRSA is primarily resistant to beta-lactam antibiotics. MRSA was originally identified in 1961 in the United Kingdom, just a year after methicillin was first employed to cure *S. aureus* infections (Enright et al., 2002, Peacock and Paterson, 2015). Regardless of the fact that methicillin is not actively used in healthcare, *S. aureus* resistance to widely used medicines such beta-lactams like oxacillin is still referred to as “methicillin-resistant.”

According to the World Health Organization (WHO) and the Centers for Disease Control and Prevention (CDC), MRSA has been a substantial and grave danger on the virulent bacteria target list, respectively(Control and Prevention, 2019, Tacconelli, 2017). MRSA alone was responsible for more than 100,000 deaths in 2019, according to a recent detailed research published in Lancet report (Murray et al., 2022). The term “hospital-acquired” or “healthcare-associated MRSA” i.e. HA-MRSA is frequently used to refer to this kind of MRSA because it was first discovered in a healthcare setting (Hibbitts and O’Leary, 2018). The illness can spread by direct contact with an inflamed wound or soiled hands. Pneumonia, Serious bloodstream infections (BSIs), sepsis and surgical site infections (SSIs) can all develop if an infection is left untreated (Siddiqui and Koirala, 2021, Kırmusaoğlu and Enany, 2017). CA-MRSA (community associated) and LA-MRSA (livestock) are additional forms of MRSA (Peacock and Paterson, 2015, Siddiqui and Koirala, 2021). Antimicrobial resistance (AMR) in the MRSA strain will be the main topic of discussion in this review. How small open reading frames (ORFs) and pan genomes of the MRSA contributes to the AMR in MRSA. Furthermore, what type of antibiotics are now used or are under study to treat MRSA infections.

## 2 Antimicrobial resistance in MRSA

The antibiotic-resistant (methicillin resistant) variant of *S. aureus* i.e. MRSA is typically resistant to beta-lactam drugs like penicillin (methicillin and oxacillin) and cephalosporin (Kırmusaoğlu and Enany, 2017, Vestergaard et al., 2019, Nandhini et al., 2022). By stopping the formation of the cell wall, beta-lactam prevents bacterial development (King et al., 2017, Pandey and Cascella, 2022, Kırmusaoğlu et al., 2019). By generating β-lactamase and changing the binding pocket for cell wall production, MRSA typically overcomes the effects of beta-lactams (Cameron et al., 2016). Various antibiotics, including teicoplanin and vancomycin, are used in the currently clinically accepted technique to treat MRSA infection. These glycopeptide antibiotics have a similar effect to beta-lactams on the bacterial cell wall, but they use a different goal by binding to the side chain of the peptidoglycan to prevent crosslinking of peptidoglycan (Ahmed and Baptiste, 2018, Stogios and Savchenko, 2020). Nevertheless, the more recent variant of MRSA began to demonstrate anti-glycopeptide drug resistance, making infection treatment challenging. Co-trimoxazole, fusidic acid, clindamycin and mupirocin are a few more antibiotics that are utilized as a second-line treatment for MRSA. However, due to the possibility of developing resistance, these antibiotics should only be used in cases where there are no other options (Brown et al., 2021, Montravers and Eckmann, 2021).

### 2.1 Heterogeneous and Homogeneous resistance of MRSA

Because of its heterogeneity, MRSA exhibits varying levels of anti-β-lactam drug resistance and other components of the culture media in which it is cultured. Only a fewer populations (1 in 106 cfu/ml) shows growth in methicillin concentrations of 5ug/ml or above conc. by showing high resistance levels, whereas the majority of cells of heterogeneous methicillin resistance (HeR) strains (99.9% or above) are susceptible to low concentrations of beta-lactam that are about 1-5ug/mL of methicillin. Homogeneous strains (HoR) can show growth in greater concentrations of methicillin that are around 5ug/mL or above and are resistant to low concentrations of beta-lactam (Chambers, 1997).

MRSA's heterogeneity is unstable and subject to alter depending on the environment for growth. When growth conditions are supplied with NaCI or sucrose to provide hypertonicity, or when larger quantities of beta-lactam antibiotic are added, or when the strains are kept at 30 °C in an incubator, HeR strains become homogenous strains (HoR). HoR strains can be transformed into HeR by adding EDTA to the growth medium or by incubating them between 37 and 43 °C. The Agr regulator system's control of gene expression is what leads to the conversion of HeR and HoR under various culture conditions (Pozzi et al., 2012). Repeated cultivations of MRSA in different media with different supplements will result in the same conversions.

In typical growth conditions, the majority of clinical variants of MRSA develop HeR, and the majority of them exhibit lower to moderate resistance levels, although a small number of subpopulations exhibit high levels of resistance (Chambers, 1997).

## 3 Role of beta-lactamase in providing AMR

By attaching to PBP (penicillin binding protein), that is in charge of GlcNAc (N-acetylglucosamine) and MurNAc (N-acetylmuramic acid) cross-linking, the cell wall of bacteria is affected by beta-lactam antibiotics. A cell wall created by this crosslinking will shield the bacteria from dangers outside the body. Pentapeptide chains typically consists of chains of l-Ala—d-Glu-l-lysine that are linked to MurNAc subunits i.e.-d-Ala-d-Ala (or -*meso*-diaminopimelic acid). Monobactams, carbapenem, penicillin and cephalosporin are examples of beta-lactam antibiotics. Their beta-lactam rings share structural similarities with the pentapeptide chain's d-Ala-d-Ala. Because of their similarities, anti-beta-lactam drugs get linked to PBP and prevent the cross-binding of glycan stands, which falls under the action of d-Ala-d-Ala substrate. Beta-lactam and binding to PBP results in the aggregation of precursors of peptidoglycan, which in turn triggers hydrolase's autolytic digestion of the old peptidoglycan. This severely compromised the structural integrity of the bacterial cell wall without the formation of fresh peptidoglycan, which causes cell injury from high internal osmotic pressure (King et al., 2017, Pandey and Cascella, 2022, Kırmusaoğlu et al., 2019).

**Fig. 1:**
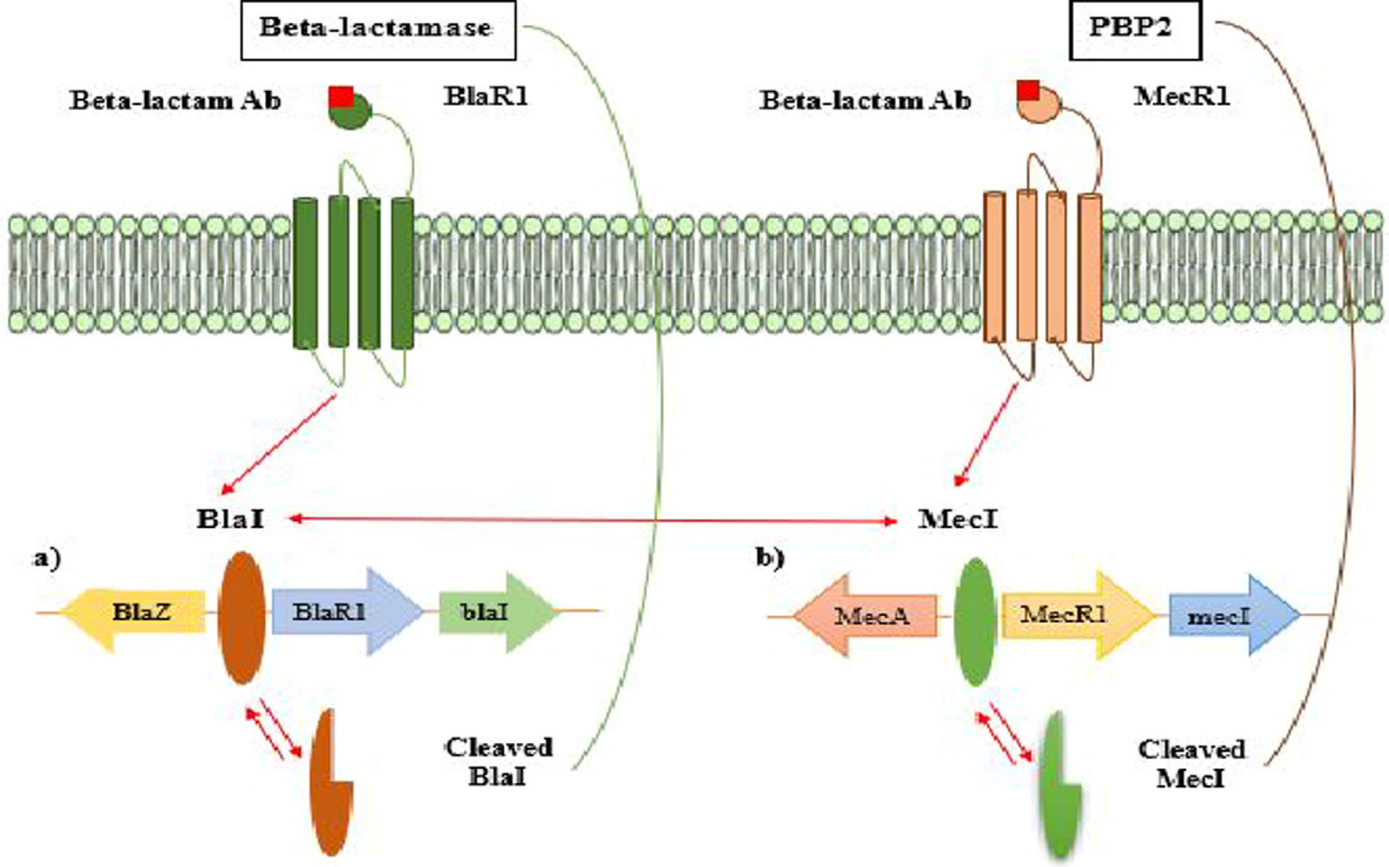
MecA and Blal's roles in MRSA resistance are correlated. a) bla operon in charge of producing beta-lactamases, and b) mec operon in charge of changing regular PBP into PBP2a. The bla and mec operons shared affinities, as shown by the blue arrows, which enables the Mec1 and Bla1 repressor to link to every operon (King et al., 2017, Pandey and Cascella, 2022, Kırmusaoğlu et al., 2019).

To overcome the unfavorable effects of beta-lactam antibiotics, MRSA produces beta-lactamase, an enzyme that breaks down the antibacterial activity of beta-lactam antibiotics, and expression of the mecA gene, which changes the penicillin-binding protein (PBP) confirmation. Bacteria manufacture the enzyme beta-lactamase to combat the effects of beta-lactam antibiotics. In the periplasmic region, this enzyme hydrolyzes beta-lactam, deactivating it prior to PBP binding (Dixit et al., 2019). The sensor protein BlaR1 and repressor Bla1 in staphylococci regulate the production of beta-lactamases (Fig. 1a) (Kapoor et al., 2017). When beta-lactam is not present, Bla1 prevents the blaZ-blaR1-Bla1 genes, which encode beta-lactamase, from transcribing beta-lactamase. BlaR1, a transmembrane sensor, binds covalently to beta-lactam once it is present and becomes acylated irreversibly at the active site containing serine. As a result, Bla1 will couple to the Bla1-blaRI operator will undergo proteolytic cleavage and will be separated from its binding pocket, activating the intracellular zinc metalloprotease domain of BlaR1. The dissociation enables the transcription of the beta-lactamase enzyme and upregulation of the blaZ gene. By preventing it from interacting with PBP, the generated beta-lactamase enzyme hydrolyzes beta-lactam antibiotics later. As a result, the bacteria can start producing peptidoglycans as usual (King et al., 2017, Pandey and Cascella, 2022, Kırmusaoğlu et al., 2019).

### 3.1 Methicillin resistance mechanism of MRSA

Mec1 and MecRI resemble Bla1 and BlaRI in terms of its morphology, mechanism, functions and molecular organization (Craft et al., 2019). Mec1 and Bla1 both control the expression of mecA. MecA is much more strongly suppressed when both Mec1 and Bla1 are available at the similar time. Since the Mec1 gene is deleted in the majority of clinical MRSA isolates, Bla1 controls mecA expression (Plata et al., 2009).

PBP2a, a new penicillin-binding protein 2a with a decreased affinity for beta-lactam antibiotics, has taken the place of the PBP that is responsible for the cross-binding of peptidoglycan in MRSA (Peacock and Paterson, 2015). The mecA gene that is present on the staphylococcal cassette chromosome mec (SCCmec), which gave rise to the resistance, can be transferred to various other subpopulations by means of horizontal gene transfer mechanism. This mecA gene cassette will be located on the MRSA chromosome once it has been acquired. Mec1 repressor and transmembrane MecR1 sensor protein regulate PBP2a synthesis (Fig. 1b). Mec1 gets attached at the promoter region of mec operon in order to suppress the mecA gene expression in the lack of anti-beta-lactam drugs (King et al., 2017, Pandey and Cascella, 2022, Kırmusaoğlu et al., 2019). Anti-Beta-lactam drugs get attached to the MecR1 sensor protein when such antibiotics are present. It activates signal transduction by prompting the metalloproteinase domain in MecR1′s cytoplasm to undergo autolytic activation. This in turn permits the expression of mecA to generate PBP2a because the latter led to the proteolytic cleavage of the Mec1 repressor from its binding site. PBP2a production permits the peptidoglycan wall synthesis to occur without the participation of beta-lactam antibiotics because of its low binding affinity to the antibiotic. It's noteworthy to notice that the mec operon and the bla operon, which produces beta-lactamase, both have a similar shape and role. Due to their similarities, the Blal repressor and the mec operon can bind and the transcription of mecA can be inhibited (Fig. 1) (Sangappa and Thiagarajan, 2012).

#### 3.1.1 Different allotypes of SCCmec machinery

On basis of *mec* genes and SCCmec elements-consisting of 3 distinct ccr (cassette chromosome recombinase) genes i.e. *ccrA, ccrB* and *ccrC*, there are 12 allotypes of SCCmec machinery identified so far. At a precise location at the 3′ end of the *rlmH* gene, each unique ccr gene contains a recombinase that regulates the incorporation and removal of SCCmec elements. It is significant to highlight that antibiotic resistance indicators, which are frequently linked to transposons, appear to accumulate in the J regions of the chromosome. There are sections encircled by IS257 that provides resistance to tetracycline [tet(K)], mercury (merAB), bleomycin (ble), aminoglycosides (aadD), as well as Tn554-like elements that encodes resistance to spectinomycin (spc) and MLS antibiotics [erm(A)] or cadmium (cad), and the last two segments are incorporated duplicates of the RC-replicating plasmids pUB110 and pT181, respectively (Partridge et al., 2018).

#### 3.1.2 Role of serene recombinase in integration of SCCmec in MRSA

The majority of SCCmec mutations encode the serine family recombinases *CcrA* and *CcrB*. Absent host-specific or SCCmec-encoded cofactors, *E. coli CcrA* and *CcrB* can perform integrative and excisive recombination. Tandem additions into the SCCmec receptor sites may be explained by *CcrA* and *CcrB's* indiscriminate substrate choice; they function on a variety of non-canonical pairs of combination sites in contrast to canonical ones. Furthermore, whereas CcrA is only necessary when one of the four half-sites is present, *CcrB* is always necessary. Recombination activity and DNA binding are correlated; *CcrA* only recognizes that portion of the recombination site that overlaps the host chromosome's conserved coding frame (Misiura et al., 2013).

### 3.2 Multidrug resistance of MRSA

Eight different forms of SCCmec exist (I-VIII). Along with methicillin resistance genes, SCCmec types II and III that exhibit multi-resistance also have genes for erythromycin and tetracycline resistance. The community-acquired MRSA strains (CA-MRSA), which are among the virulent infections and infect healthy people outside of hospitals, require SCCmec type IV. Other varieties of SCCmec are uncommon(Otto, 2010).

By cloning and analyzing the sequences of SCCmec components from different MRSA strains around the world, we discovered that SCCmec can take on a variety of allotypic forms. There are two crucial genetic components that make up SCCmec. The first component is the mec gene complex, which consists of the regulatory genes *mecR1* and *mecI* and the complete or trimmed sets of *IS431mec* and *mecA*. As far as our testing goes, *mec* gene complexes can be divided into four groups based on their structural characteristics:

1. Class A: IS431-mecA-mecR1-mecI,
2. Class B: IS431-mecA-mecR1-IS1272,
3. Class C: IS431-mecA-mecR1-IS431, and
4. Class D: IS431-mecA-mecR1.

The *ccr* gene complex, which consists of the 2 site-specific recombinase genes *ccrA* and *ccrB* that are in charge of SCCmec's mobility as well as nearby orfs with unknown functions, is the second crucial area (Ito et al., 2003).

**Staphylococcus IS431** is mostly present in chromosomes and plasmids and is associated with encoding a number of resistance factors, including tetracycline, mercury, and cadmium resistance. Methicillin-resistant Staphylococcus can develop multiple drug resistance if other additional resistance genes, such as *aadD* encoding an enzyme for tobramycin resistance, are integrated inside SCCmec cassette (IS431mec). Additional resistance genes generating bleomycin resistance (ant(4′)), tobramycin, kanamycin, heavy metals and penicillin resistance, and tetracycline resistance are included in the plasmids pUB110, pI258, and pT181 incorporated in SCCmec. Additional resistance gene *ermA*, which encodes inducible streptogramin, macrolide and lincosamide resistance, is present in the SCCmec-integrated Tn554 (Otto, 2010).

## 4 SNPs in mprF ORF provides daptomycin resistance in MRSA

MprF lysinylates phosphatidyl-glycerol (PG) to create the positively charged phospholipid (PL) species, lysyl-PG (L-PG). It has been proposed that the Single nucleotide polymorphisms (SNPs) discovered within the mprF ORFs are connected to a gain-in-function phenotype in terms of daptomycin resistance in MRSA. Daptomycin (DAP) has been used in numerous therapeutic contexts since the FDA approved it in 2003, particularly for resistant MRSA infestations including endocarditis (Rose et al., 2007, Schriever et al., 2005, Tally and DeBruin, 2000). There have been several recent reports of clinical MRSA variants that have developed in vitro DAP resistance in the setting of failing DAP treatment regimens, particularly in endovascular syndromes (Bayer et al., 2013, Boucher and Sakoulas, 2007, Humphries et al., 2013, Jones et al., 2008).

One of the primary hallmarks of DAP-resistant (DAPr) MRSA variants is gain-in-function mutations in a very small number of genes, mainly in the mprF region (Bayer et al., 2014, Mishra et al., 2013, Pillai et al., 2007). The MprF protein synthesizes the specific positively charged phospholipid (PL) lysyl-phosphotidylglycerol (L-PG), which is then translocated (flipped) from the inner to outer cell membrane (CM) leaflet (Ernst et al., 2009, Weidenmaier et al., 2005). L-PG production is wholly dependent on the lys-tRNA binding site found in the C-terminal cytoplasmic domain (Ernst et al., 2015). Numerous researchers have proposed that an increase in L-PG synthesis and flipping causes a rise in positive surface charge, which produces a charge-repulsive environment for cationic molecules including host defense peptides (HDPs) and calcium-complexed DAP (Yang et al., 2013, Yang et al., 2010). Several laboratories have established a connection between the DAPr phenotype and the existence of SNPs inside the mprF gene throughout the course of the previous few years (Bertsche et al., 2013, Mishra et al., 2014). The mprF open reading frame (ORF) contains all of these SNPs, but there are obviously hotspots for those SNPs associated with DAP resistance within this locus (Bertsche et al., 2013).

**Fig. 2:**
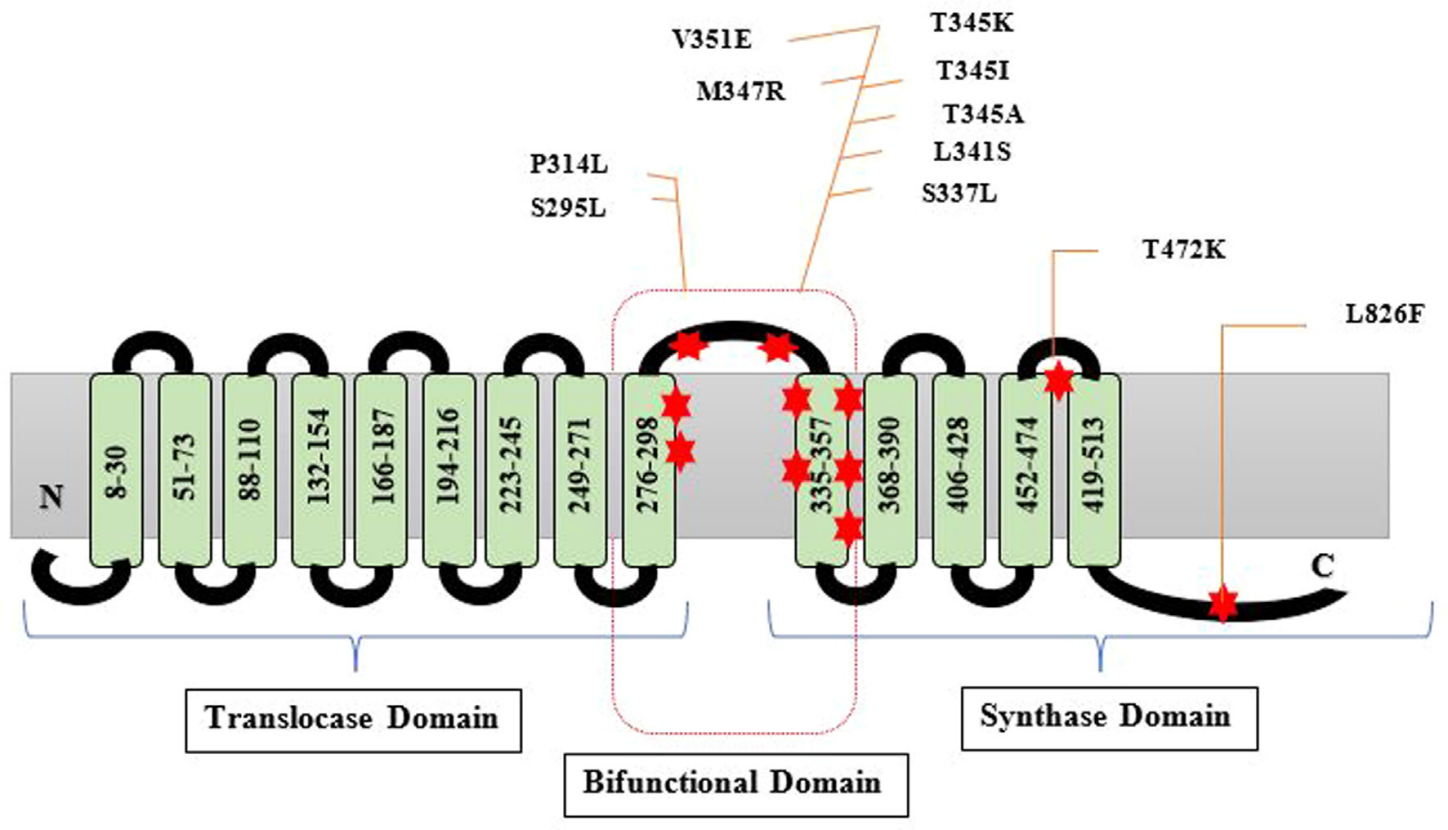
Locating specific mutations in the MprF protein. A dual-purpose enzyme called the *S. aureus* multiple peptide resistance factor (MprF) aids positively charged L-PG in moving from the inner to outer leaflet of the CM. A core bifunctional domain, a C-terminal L-PG synthase domain, and an N-terminal L-PG translocase (flippase) domain make up MprF. This domain connects the L-PG synthase and translocase domains at either end. Compared to past efforts, the projected MprF topology was different (Bayer et al., 2014).

This hinderance was addressed in a study by Bayer and his associates using 22 clinical methicillin-resistant *S. aureus* (MRSA) strain combinations that were daptomycin-susceptible (DAPs)/daptomycin-resistant (DAPr). It was discovered that the transmembrane tract around the synthase-translocase interface included amino acid changes in 12 out of 22 DAPr isolates, or about 55% of the DAPr strains (central bifunctional domain). Seven out of the twenty-two DAPr isolates (or about 32% of the DAPr strains) had the amino acid sequence changes inside the synthase domain of MprF. Most of the mprF SNPs detected in DAPr strains were found to be localized inside the two MprF loci, based on the locations of the most frequent changes (Fig. 2) i.e.

(i) the central bifunctional domain and
(ii) the C-terminal synthase domain

Additionally, they were able to correlate the presence and positioning of *mprF* SNPs in DAPr strains with HDP cross-resistance, positive surface charge, and L-PG profiles. Despite the fact that DAPr strains with *mprF* SNPs in the bifunctional domain had higher resistance to *tPMPs* than DAPr strains with SNPs in the synthase domain, positive surface charge tests failed to detect this connection. These results demonstrated that both charge-related and charge-unrelated pathways in *S. aureus* mediate DAP resistance and HDP cross-resistance (Bayer et al., 2015).

## 5 Newly acquired linezolid resistance in MRSA

The first oxazolidinone class antibiotic to be approved is linezolid (LZD). MRSA and other infectious disorders brought on by Gram-positive bacteria can be effectively treated with this medication. By attaching to 23S rRNA domain V, LZD prevents the assembly of the essential 70S initiation complex that is needed for translation of bacterial proteins, hence inhibiting protein synthesis (Biedenbach et al., 2010). There have been several documented processes of LZD resistance, including mutations of the ribosomal proteins near the binding pocket of linezolid in the central core of ribosomal peptidyl transferases (*rplV, rplD, and rplC*) (Locke et al., 2009), procurement of *fexA* or *cfr* that encodes a 23SrRNA methylase (Toh et al., 2007)and point mutations of the 23S rRNA gene's domain V (G2447T, C2461T, T2500A, or G2576T ) (Wilson et al., 2003).

LZD resistance, on the other hand, only occasionally manifests clinically and has only ever been noted after a protracted drug exposure (Meka et al., 2004). This finding is in line with the low reported in vitro mutation frequency of less than 8 1011 (Brickner et al., 1996). After only two weeks of LZD treatment, a study recently revealed the isolation of multiple LZD-resistant *S. aureus* (LRSA) strains from patient blood cultures (Perlaza-Jiménez et al., 2022). Following exposure, LZD's minimum inhibitory concentration (MIC) was 32 g/ml as opposed to the parent strain's MIC of 4 g/ml. The G2576T mutation was present at several 23S rRNA loci in several LRSA strains. While there is no explanation for why these strains acquired LZD resistance so quickly, the impact is evocative of a hypermutator phenotype. High point mutation frequencies are present in hypermutator bacteria, frequently as a result of the turning off of repair genes that are mismatched, hastening the development of AMR (Jolivet-Gougeon et al., 2011). The distribution of mutations of domain V among the many copies of 23S rRNA genes in a particular N315 genome has been linked to homologous recombination, according to another research (Lobritz et al., 2003, Boumghar-Bourtchaï et al., 2009).

Iguhci and his colleagues carried out a study to examine 23SrRNA loci, including V domain and 16S-23S intergenic spacer regions and SNPs, in an effort to understand the process of the fast acquiring of linezolid resistance in the clinical variants of MRSA. It was discovered that the priorly exposed variant (M2) acquired linezolid resistance much more quickly than N315 and that variants that are obtained from the subjects before exposure to linezolid (M5-M7) displayed greater levels of LZD resistance as compared to N315. G2576T mutations were the most prevalent method of linezolid resistance in 40 of 63 (63.5%) LRSA strains (Gu et al., 2013). Contrary to the formation of LZD resistance after LZD administration for 20–48.9 months, After just two weeks of clinical exposure to LZD, these clinical LRSA strains developed the often reported rrn mutation (G2576T) in three rrns (Endimiani et al., 2011). However, the mutation rates of these and other clinical isolates from the same time period were equivalent to those of N315, and the isolates lacked any changes in genes associated with hypermutation. Clinical strains from both before and after exposure shared the same sequences for the 16S-23S intergenic spacer region and the 23S rRNA gene. In all the pre-exposure isolates, a recQ missense mutation (Glu69Asp) with reference to N315 was found, which is noteworthy. This lesion might have impaired short sequence recombination. This mechanism was thought to have accelerated the development of linezolid resistance (Iguchi et al., 2016).

### 5.1 Conjugated transfer of cfr resistance genes plasmid to MRSA

A clinical MRSA strain from Colombia in 2005 was the first human isolate to have the multi-resistance gene *cfr*, which was first discovered in a bovine *Mammaliicoccus sciuri* strain in 2000 from Germany It's interesting to notice that *S. capitis* had the highest prevalence of the pLRSA417-like plasmids among the clinical Staphylococcus species isolated from humans. Additionally, the fact that pLRSA417-like vectors were susceptible of interspecies transmission through genetic drift even in vivo was demonstrated by the fact that the homologous plasmids pTZ273 and TZ266 from *S. cohnii* TZ273 and pTZ266 from *S. capitis* originated from a single patient. The capability of pLRSA417-like plasmids for conjugative transfer was demonstrated by a study.

S. aureus RN4220NR and Enterococcus faecalis JH2-2 recipient strains could accept S. aureus plasmids pTZ41, pTZ41, pTZ24, pTZ99, pTZ273, and pTZ273 from human clinical isolates. Additionally, the recipient strain was S. capitis TZ24, which lost the cfr-bearing plasmid in serial passage (named TZ249), and the donor strains were the transconjugants RN4220-cfr and JH2-2-cfr. Nearly 10–3 transconjugants were obtained per receiver, which is a transfer frequency. These findings demonstrated the plasmids' capacity for intraspecies and cross species conjugation as well as S. capitis TZ249′s capacity to ingest pLRSA417-like plasmids (Gao et al., 2022).

### 5.2 23S rRNA gene polymorphisms within chromosomes

The 23S rRNA genes of MRSA are present in 5 or 6 copies and exhibit numerous intrachromosomal sequence variants (Table 1). The 23S rRNA genes in one copy of M2 and M3 were found to have 3 SNPs; the other four rrn loci in each of the genome contained sequences that were the same as each other and the reference genome. Particularly, in comparison to the N315 genome, rrn2 contained the following substitutions i.e. T1584A, C280A and C441T. The rrn sequences of strain M2/128/15 (parent variant/MIC/passage number, i.e., a strain developed from M2 after 15 passes with LZD) were similar to those of the parent strain, with the exception of the presence (in all 5 loci) of a G2576T mutation (Iguchi et al., 2016, Maarouf et al., 2021).

**Table 1:** 23S rRNA gene polymorphisms within chromosomes.

**Wild type strain**	**23S rRNA gene polymorphisms within chromosomes**										
	**2576**	**2261**	**1584**	**441**	**428**	**312**	**307**	**280**	**261**	**223**	**48**
**M2/128/15**^b^	T (rrn1 ∼ 5)	G	T,A (rrn2)^a^	C,T (rrn2)^a^	G	A	A	C,A (rrn1,2)^a^	C	G	G
**M2, M3**	G	G	T,A (rrn2)^a^	C,T (rrn2)^a^	G	A	A	C,A (rrn1,2)^a^	C	G	G
**Mu50**	G	G,A (rrn1)^a^	T,A (rrn1,2)^a^	C	G	A,G (rrn3)^a^	A,G (rrn2)^a^	C,A (rrn1,2)^a^	C	G	G
**N315**	G	G,A (rrn1)^a^	T,A (rrn1,2)^a^	C	G	A,G (rrn1)^a^	A	C,A (rrn1,2)^a^	C	G	G

(Iguchi et al., 2016, Maarouf et al., 2021).

a Majority and the minority base.

b parent variant/Linezolid MIC/passage no.

### 5.3 Parent strain traits and isogenic mutant traits discovered using stepwise LZD resistance selection tests

The table below illustrates the correlation between the minimum inhibitory concentrations of linezolid in variants produced through step by step selection and the copy number of the mutant domain V gene in 23S rRNA. All of the rrn loci in M2/128/15 contained the E. coli numbering type of mutation G2576T, while three of the rrn loci in M3/128/32 had the G2447T type of mutation. As fewer LZD-resistant mutants were chosen step by step from our clinical variants, the number of domain V mutation copies tended to increase. However, only one of the rrn loci in the LZD-resistant mutants created by LZD selection from M13 or N315 strains included the G2236T (MRSA numbering) mutation (Iguchi et al., 2016, Maarouf et al., 2021).

### 5.4 Involvement of VITEK 2 system in providing linezolid resistance to MRSA

The VITEK 2, a computerized susceptibility testing method, undergoes quick determination of minimum inhibitory concentrations (MICs) through the examination of the growing dynamics of bacteria in test cards treated with antibiotics. Standardized interpretive readings of these MICs are provided by the Advanced Experts system (AES). In a nutshell, it consists of a database of MIC concentration for various antibiotic combinations and common resistance mechanisms in many species, together with a number of algorithms. The VITEK 2 isolate's MIC phenotype is compared to every pattern in the database, and the best fit is determined. Users are informed when the estimated framework anticipates clinical resistance to medications to which the bacteria seemed susceptible at breakpoint, and they are also informed when unusual pairings of phenotype and species are noted and warned against (Livermore et al., 2002) (see Table 2).

**Table 2:** MIC of LZD for 23SrRNA gene copies carrying the Domain V mutation in LZD-resistant and -sensitive strains.

**Wild type strain**	**Mutational site**	**MIC of LZD for 23SrRNA gene copies carrying the Domain V mutation in LZD-resistant and -sensitive strains**					
		**128**	**64**	**32**	**16**	**8**	**4**
**N315**	G2236T	rrn1,2,3,4,5(15)^b^		rrn1,3,4,5(10)^b^		rrn1,4,5(7)^b^	ND^a^ (6)^b^
**M13**	G2236T	rrn3,4,5(32)^b^	rrn1,4,5(29)^b^	ND^a^ (15)^b^	ND^a^ (8)^b^	ND^a^ (6)^b^	
**M2**	G2447T				rrn1(32)^b^	rrn1(8)^b^	rrn1(6)^b^
**M3**	G2576T				rrn1(32)^b^	rrn1(10)^b^	rrn1(7)^b^

(Iguchi et al., 2016, Maarouf et al., 2021).

a ND = no domain V mutation detected.

b = passage.

Utilizing the VITEK 2 technology, the clinical traits of MRSA having the enhanced minimum inhibitory concentrations of LZD in light of the numerous LZD resistance processes have been discovered in MRSA. The presence of the cfr(B), cfr and optrA genes, as well as alterations in the 23S rRNA gene or ribosomal proteins (L22, L3, and L4), were all evaluated by using the broth microdilution (BMD) test on 27 of the MRSA variants from fourteen subjects with MICs 8 g/ml of LZD. Four (14.8%) of the 27 variants from 1 subject were found to be LZD-resistant shown by BMD and have the T2500A mutation in 23S rRNA gene. The linezolid-resistant results were significant VITEK 2 mistakes because the remaining 23 were proven to be linezolid sensitive. L3 Gly152Asp was the most often found mutation (19/27, 70.4%), and it was exclusively found in isolates that were susceptible to the drug linezolid. No isolates had any L4 or L22 protein changes, optrA, cfr, or cfr(B). The outcomes demonstrate that the L3 Gly152Asp mutation was not significantly associated with LZD resistance, however the 23S rRNA T2500A mutation was. For VITEK 2 LZD-resistant findings, a confirmatory test is indicated due to the possibility of false-positive results (Yoo et al., 2020).

## 6 The transmission of mecA, a methicillin resistant gene, from MRSA to MSSA

The incidence of broad antibiotic drug use has increased antimicrobial resistance at the same time. It has been noted that the rise in the incidence of microorganisms that are resistant to antibiotics poses a serious danger to the treatment of infectious diseases. Mobile genetic elements (Table 3) with genomic islands, such as conjugative plasmids and transposons, which are known to facilitate the horizontal gene transfer of resistance genes to other bacteria, have been shown to influence the development of resistance. Concern over MRSA's development as a frequent source of infections acquired in hospitals (HA) is growing. This is due to the fact that the majority of MRSA strains have acquired multiple resistant determinants like conjugation plasmids that carry resistance against gentamicin, to achieve their multi-resistant status (Ohlsen et al., 2003).

**Table 3:** Mobile genetic elements (MGEs) detected in MRSA.

**MGE**	**Description**	**Examples**	**Explanation**
Toxicogenic Bacteriophages	Lytic: Bacterial lysis Temperate: Ongoing interaction with cells Chronic: Generate progeny without destroying the host	SEA (Staphylococcal enterotoxin A), Luk-PV (PVL), CHIP (chemotaxis inhibitory protein)staphylokinase and SCIN (Staphylococcal complement inhibitor)	Have largest impact on *S. aureus* diversity and evolution and appears to be widespread in *S. aureus* (1–4) per strain
Transposons and plasmids	Toxins, arginine metabolism, heavy metal, antibiotic, and disinfectant resistance determinants are carried by transposons (Tn552) and plasmids (small and big).	Based on physical/genetic organization and functional properties, plasmids can be divided into four classes: pSK639 family, small multicopy; pSK41, conjugative, big plasmids	
SCC (non-mec)	Encoding methicillin resistance is not the only option.	SCC mercury	Provides resistance against mercury chloride
Staphylococcal cassettechromosome mec (SCCmec)	SCCmec are substantial DNA strands that *S. aureus* inserts into the orfX gene. Type 1 RM systems for Sau1	Types (I-XI) SCCmec	MecA, a gene for meticillin resistance, is encoded by SCCs, can encode factors of virulence and/or antibiotic resistance.
Genomic islands		vSA, vSA, and vSA are three families.Responsible for the formation of bacteriocin, enterotoxins, phenol-soluble modulins (PSMs) , and maybe pro-inflammatory activities.	A defective transposase gene upstream and an incomplete type 1 RM system downstream
Pathogenicity and compositeislands (SaPIs)	Similar to phages but lacking the genes needed to build the capsid heads and tails required for horizontal transfer. encode TSST or enterotoxins	Produce genes that encode TSST, superantigens (SEB, SEC), MDR transporters, and fusidic acid resistance	≥16 sequenced. 0–2 per strain 14–17 kb in size

RM, restriction modification, MDR, multidrug resistance; TSST, toxic shock syndrome toxin; and PVL, Panton–Valentine leucocidin.

A gene termed cassette chromosome recombinase (ccr) promotes the site-specific administration of the SCCmec into MRSA genome at a location known as the SCCmec attachment or insertion area. (Berger-Bächi and Rohrer, 2002, Barlow, 2009). These recombinant gene work in form of dimers, and in case of ccrAB gene, they make it easier for SCCmec to be incorporated into the chromosomal of *S. aureus*. It is attached to the core recognition sites (attB and attSCC), one on the MRSA chromosome and the other on the mec gene machinery itself, to accomplish this. The ORFs of unknown origin (orfX), also known as the universal administration area for SCCmec, has a 15 base pair sequence called attB which is located on the chromosomal end (Wang and Archer, 2010).

At either end of the SCCmec, two hybrid sites known as the attL and attR are formed as a result of the SCCmec's integration into the genome (Wang and Archer, 2010). It is widely known that methicillin-susceptible *S. aureus* (MSSA) changed into MRSA after acquiring the genomic island containing the mecA methicillin resistance determinant (Chongtrakool et al., 2006). Therefore, a study was created to explain the transfer mecA mechanism in detail as well as to pinpoint its evolutionary origin (Hanssen and Ericson Sollid, 2006, Aklilu et al., 2013). In vitro transmission of the mecA methicillin resistance gene to MSSA. The recipient transconjugants were mecA positive and resistant to erythromycin and cefpodoxime. Following mix culture plating on Luria Bertani agar containing 100 ug/mL, PCR amplification of the mecA gene revealed that 75.5% of the donor and 58.3% of the recipient transconjugants were mecA positive. Furthermore, 41.75% and 46.2% of recipient and donor *trans*-conjugants were mecA positive on LB agar containing 30 ug/mL and 50ug/mL respectively, while 61.5% of recipient *trans*-conjugants and donor cells were mecA positive (Bitrus et al., 2017).

## 7 Pangenome analysis of MRSA strains and their role in AMR

The pan-genome, which consists of a core genome, which contains sequences shared by every member of the species, and a “dispensable” genome, represents every gene in a species. In 2005, the genomes of 6 variants of S. agalactiae were mapped, indicating a core genome including 80% of S. agalactiae genetic makeup. This was the first time the concept of a pan-genome was proposed for bacterial species. Since then, attempts have been made to understand how various bacterial species' pan-genomes contribute to the development of antibiotic resistance (Tettelin et al., 2005).

62 distinct sequence types (STs) of *S. aureus* were found in epidemiological studies, with ST-8, ST-5, ST-398, ST-239, and ST-30 being the most prevalent STs and accounting for more than 50% of the isolates. 7,199 genes remain unannotated in the *S. aureus* pan-genome, which contains a significant (80%) contribution from MRSA strains. The 2,025-gene core genome of *S. aureus*, which makes up 72% of the whole genome size, is essentially steady, while the number of auxiliary and unique genes (28% of the total genome size) is gradually rising (Naz et al., 2022).

### 7.1 Microevolution and pangenome analysis of USA300 MRSA

A prominent source of skin and soft tissue infections in the United States is the *S. aureus* clone MRSA strain USA300 (SSTIs). MRSA infections were widespread in the United States in the 1990 s outside of medical facilities (King et al., 2006). By 2004, more than 97% of MRSA isolates from community-associated SSTIs in the US belonged to the USA300  (Alam et al., 2015).

In an early whole-genome sequence (WGS) analysis of a single USA300 strain, a Staphylococcal Cassette Chromosome mec (SCCmec) type IVa element, a *S. aureus* pathogenicity island 5 (SaPI5), a Panton-Valentine leukocidin-carrying Sa2 prophage, and an arginine catabolic mobile element (ACME) type I element were found (Diep et al., 2008). These 4 MGEs serve as USA300′s most prevalent molecular markers (Uhlemann et al., 2014)A reference plasmid, pUSA300HOUMR, in the size of 27 kb annotated through bioinformatic tools like BRIG or Snap gene with genes that give resistance to erythromycin (mphBM and msrA), kanamycin (aphA-3), penicillin (blaZ), bacitracin (bcrA) (Kennedy et al., 2010), 3.1 kb cryptic plasmid and streptothricin (sat) are also frequently found in USA300 isolates (Kennedy et al., 2010). The role of MGEs in USA300′s success has been disputed, leading to the suggestion that the accessory genome has encouraged USA300′s improved transmission. In particular, it has been hypothesized that the ACME element enhances USA300 survival on the skin of human and is found to be persistent within the cutaneous abscesses (Thurlow et al., 2013). In contrast, modest genetic alterations within the core genome as well as altered expression of key virulence determinants including alpha-toxins, an AGR (accessory gene regulator) and PSMs (phenol-soluble modulins) may have played a role in the pathogenesis of USA300 (Li et al., 2009). Core genome SNPs could also cause USA300 isolates to exhibit noticeably distinct virulence characteristics because to variations in exoprotein expression (Kennedy et al., 2010).

Reconstructing evolutionary relationships showed that USA300 isolates (154/191, 81%) predominated in CC8, which was diverse and showed little phylogeographic clustering. The USA300 auxiliary genome was essentially homogenous and made up of components that had previously been connected to this lineage. In more recent samples, SCCmec -ve and ACME -ve USA300 variants started to appear, and the prevalence of the Sa5 prophage increased. Due to enhanced heterogeneity in core genome and temporary alterations in the frequency of specific accessory elements, the analyzed *S. aureus* USA300 collection as a whole revealed an evolving pan-genome (Jamrozy et al., 2016).

### 7.2 Pangenome analysis of MRSA CC398 isolates

The clonal complex (CC) 398, which can lead to typical MRSA-associated infections in humans, is one of the most significant livestock-associated MRSA genetic lineages. In one study, bioinformatics analysis was used to ascertain the genetic properties of three MRSA CC398 isolates acquired from humans (strains C5621 and C9017) and an animal (strain OR418). Of the three strains, C9017 shown the widest genotype of resistance, including resistance to the antimicrobial classes of aminoglycoside, fluoroquinolone, clindamycin, macrolide and tiamulin. The immune evasion cluster system's scn, sak, and chp genes were only found in OR418. Total 288 specific genes of newly isolated variants were discovered by pangenome analysis, the majority of which are hypothetical or connected to phages. The strongest genetic changes were seen in OR418. In OR418 and C5621, the RNAIII (-hemolysin) gene was without a doubt the most expressed gene, however in C9017, it was not found. The proteome profiles of the several strains revealed significant variations. For instance, OR418 has higher levels of the Sbi (which is an immunoglobulin-binding protein). The findings suggest that the OR418 strain has a high potential for zoonotic transmission since Sbi is a multifunctional immune evasion component in *S. aureus* (Ribeiro et al., 2022).

### 7.3 Pangenome analysis of MRSA CC130 isolates

Several MRSA lineages, primarily those connected to animals, including CC130, CC49, ST425, CC599, and CC1943, have been identified to contain the mecC-gene. The most frequent mecC lineage is CC130, which is related with ruminants (Paterson et al., 2014, Zarazaga et al., 2018). Due to its recovery from a variety of animal hosts and the fact that it lacks the immune evasion cluster (IEC) that is peculiar to humans, this lineage has historically been thought to be animal-associated. However, occasional mecC-MRSA human infections have been documented (Lozano et al., 2020), with some instances demonstrating zoonotic transmission (Harrison et al., 2017).

This mecC-MRSA-CC130 lineage lacks significant human virulence characteristics and appears to be responsive to a variety of non-lactam medications (Monecke et al., 2016, Cuny et al., 2015). They do, however, carry a unique allele of the exfoliative toxin gene (called etd2), which may account for the diverse range of hosts. Mobile genetic elements (MGEs) or chromosomal alterations may be linked to *S. aureus*' adaptation to specific host species. A sign for some level of human host adaptability is thought to be the genes of the human specific immune evasion cluster (IEC), in particular. Based on the combination of five genes (scn, chp, sak, sea/sep), this IEC system is found in seven different configurations (types A–G); the scn gene (encodes a staphylococcal complement inhibitor) is included in all IEC types, is frequently used as a marker of IEC-positive isolates and is functionally essential (van Wamel et al., 2006). Except for a few isolates from ST1945, ST1581, and ST1583 previously described by our group from wild animals and intensively farmed domestic animals (Ruiz-Ripa et al., 2019, Gómez et al., 2016, Gómez et al., 2015)and one ST1945 MRSA strain from a human sample, none of the mecC-MRSA reported strains carried the scn gene (important for the IEC system) (Harrison et al., 2017).

MRSA-CC130 was identified to contain antibiotic resistance genes connected to the SSCmecXI element. The majority of MRSA-CC130 strains shared a set of virulence genes. Additionally, one MSSA-ST130 and six MRSA-CC130 both had lukMF'. In terms of resistance and virulence genes, the MSSA-ST700 strains differed the most. The pan-genome study revealed that 21 MSSA-CC130 isolates and 29 genes unique to MRSA-CC130 (related with SCCmecXI) were detected (associated with phages). SCCmecXI, PBP3, GdpP, and AcrB were identical across all strains in terms of amino acids, however PBP1, PBP2, PBP4, and YjbH proteins showed some variation (Gómez et al., 2021).

### 7.4 Pangenome analysis of MRSA with heterogenous intermediate resilience to vancomycin (hVISA)

VISA (Vancomycin-intermediate Staphylococcus aureus) cells are produced spontaneously by the hVISA isolates through a variety of fascinating processes. In one study, the core genomes of 39 such hVISA strains were analysed. The MRSA linked with the hVISA phenotype's genomic relatedness was not found. In 92% of the hVISA, the alteration Try38 to His in Atl (autolysin) was found. In 11 coding areas with putative roles in virulence, transport systems, carbohydrate metabolism, and tRNA synthesis, we found SNPs and k-mers linked to hVISA. Additionally, genes for lacABCDEFG were downregulated in hVISA whereas genes for capABCDE, sdrD, esaA, esaD, essA, and ssaA were overexpressed. Additionally, whilst arginine, glycine, and betaine were more plentiful in hVISA, valine, threonine, leucine tyrosine, FAD, and NADH were more abundant in VSSA. The purine and pyrimidine route, CoA biosynthesis, amino acid metabolism, and aminoacyl tRNA biosynthesis were among the altered metabolic pathways that was seen in hVISA (Castro et al., 2022).

Similar results were found in another investigation when a total of 39 MRSA isolates (3.3%) were identified as hVISA (1.4% of MRSA recovered from 2006 to 2008 and 5.6% from 2011 to 2014). 95% of the hVISA strains belonged to clonal complex (CC) 5, which is the largest. Only 6/39 hVISA isolates were classified as such by PAP/AUC, with 6 additional isolates falling within the cut-off range (0.87 to 0.89). (0.9). The Leu-14-Ile (90%) and VraT Glu-156-Gly (90%) amino acid changes in WalK were present in most of the 39 hVISA isolates. Additionally, we discovered 10 alterations in the proteins WalK, VraS, RpoB, and RpoC that are exclusive to hVISA isolates (Castro et al., 2020).

## 8 Core-genome Multi-locus sequence typing (MLST) to evaluate MRSA epidemiology

Although the molecular mechanisms underpinning the development of CA-MRSA are not entirely understood, during the past 20 years, numerous investigations have observed alterations in the epidemiology of MRSA (David et al., 2014). Increasing numbers of patients in hospital settings have been seen with MRSA infections brought on by lineages that were previously only linked to CA-MRSA (Otter and French, 2006). In a hospital in Shanghai from 2005 to 2014, Li and his colleagues reported an increase in CA-MRSA infections brought on by Panton-Valentine (PVL) leukocidin-negative ST59 MRSA (Li et al., 2016). In another study, Li with his colleagues examined 416 MRSA bacteremia isolates from 22 teaching institutions and discovered that, between 2013 and 2016, ST59 isolates predominated over ST239 isolates across all MRSA cases. However, it is unclear what causes these epidemiological shifts (Li et al., 2018).

A study is being conducted by Chen and his colleagues to clarify how MRSA's epidemiology is changing. The research led to the discovery of 292 MRSA infections. Sequence type (ST) 5 accounted for the bulk of them (51.4%), followed by ST59 (23.3%; 68 of 292). ST5 MRSA's percentage decreased from 68.3% to 32.1% in 2015, whereas ST59 MRSA's percentage increased from 8.9% to 41.0%. Core-genome phylogenetic analysis revealed that ST59 MRSA isolates had more genotypic diversity than ST5 MRSA isolates in both the healthcare-onset and community-onset categories. According to minimum spanning trees, SRRSH had a cluster of ST5 MRSA infections while most ST59 MRSA infections were scattered. The ST59 MRSA strain, which caused 45.2% of the 93 skin and soft tissue infection cases, exhibited lower rates of levofloxacin (11.8%) and ciprofloxacin (19.1%) resistance than the ST239 and ST5 MRSA isolates. Higher virulence was found in ST59 healthcare-onset MRSA in both the cutaneous infection model and the hemolysis assays. Notably, the levels of pathogenicity of these isolates were comparable to those of common MRSA strains found in the population (Chen et al., 2018).

### 8.1 Antimicrobial resistance

Multidrug resistance was common among MRSA isolates as expected, however ST59 MRSA isolates displayed a distinct resistance profile than ST5 and ST239 MRSA (Table 4). Levofloxacin (8 of 68; 11.8%) and ciprofloxacin (13 of 68; 19.1%) resistance rates were modest in the ST59 MRSA isolates, but they were nearly universal in the ST239 and ST5 isolates. Linezolid was not effective against one ST5 MRSA isolate. Vancomycin and tigecycline were both ineffective against any of the MRSA strains. These MRSA isolates showed high levels of clindamycin resistance. The percentages of MRSA ST5, ST59, and ST239 isolates that were resistant to clindamycin were greater than 75% (Chen et al., 2018).

**Table 4:** MRSA Isolate AMR Profiles from SRRSH, 2013–2015.

**Antimicrobial**	**Resistant isolates, No. (%)**		
	**ST239 (n = 19)**	**ST5 (n = 150)**	**ST59 (n = 68)**
**VAN**	0 (0)	0 (0)	0 (0)
**LNZ**	0 (0)	1 (0.7)	0 (0)
**TGC**	0 (0)	0 (0)	0 (0)
**TCY**	18 (94.7)	104 (69.3)	23 (33.8)
**GEN**	18 (94.7)	6 (4)	0 (0)
**RIF**	13 (68.4)	0 (0)	1 (1.5)
**SMZ**	6 (31.6)	1 (0.7)	0 (0)
**CLI**	15 (78.9)	148 (98.7)	60 (88.2)
**ERY**	17 (89.5)	149 (99.3)	61(89.7)
**CIP**	19 (1 0 0)	149 (99.3)	13 (19.1)
**LEV**	19 (1 0 0)	149 (99.3)	8 (11.8)

Abbreviations: CIP, or ciprofloxacin; CLI, or clindamycin; ERY, or erythromycin; GEN, or gentamicin; LEV, or levofloxacin; TCY, tetracycline; TGC, tigecycline; VAN, vancomycin, SMZ, or trimethoprim-sulfamethoxazole; LNZ, linezolid; SRRSH, or Sir Run Run Shaw Hospital; ST, or sequence.

## 9 Mrsa's core genome allelic patterns for predicting resistance to penicillin and potassium clavulanate

The three medications that are most frequently prescribed to treat MRSA infections are vancomycin, daptomycin, and linezolid (Dixit et al., 2019). The likelihood of vancomycin failing to treat MRSA infections is quite high due to its limited tissue penetration, sluggish bacterial clearance, and side effects. Daptomycin and linezolid have been shown in prior research to have numerous drawbacks in curing MRSA diseases, that includes the development of resilience and significant adverse impacts (Lv et al., 2021). The restoration of beta-lactam susceptibility in MRSA has recently been studied using a variety of methods, including the simultaneous injection of beta-lactamase inhibitors such clavulanic acid. Harrison's in vitro and in vivo research revealed that mutations in the mecA genes and their promoters caused a significant portion of MRSA from different lineages to be susceptible to penicillin plus 15 mg/l potassium clavulanate (PENC) (Murray et al., 2022).

It was discovered that PENC may be effective in treating some MRSA infections. In order to predict medication susceptibility for the PENC phenotype, the MRSA variant’s susceptibility was examined. The disadvantage of using a single component to predict drug susceptibility was highlighted by the discovery that S2-R isolates with susceptible mecA genotypes but PENC-resistant phenotypes expressed at higher levels than S2 MRSA (2.61 vs. 0.98). The data of selected UK-sourced MRSA (n = 74) and MRSA gathered in a previous national survey (NA-MRSA, n = 471) were used as the training sets to create a model with accuracy of 0.94 and 0.93 for SRRSH-MRSA and UK-sourced MRSA (n = 287, NAM-MRSA) validation sets, respectively. The AUROC of this model was 0.96 for SRRSH-MRSA and 0.97 for NAM-MRSA, respectively. Despite the fact that the source of the training set data affects the prediction model's scope of applications, the data demonstrated the efficacy of the machine learning approach in predicting susceptibility from cgMLST findings (Zhuang et al., 2021).

## 10 Conclusion

MRSA poses a serious hazard to human health because it can become resistant to any antibiotic to which it is subjected. While antibiotics have only been used clinically for about 80 years, bacteria have long since developed resistance mechanisms. As the usage of antibiotics has increased, bacteria have had to adapt and develop sophisticated ways to live. Different SNPs and mutations in genome of MRSA causes it to develop resistance against long used antibiotics used for MRSA treatment like daptomycin, vancomycin etc. Core and pan-genome analysis of these antibiotic resistant MRSA strains helps scientist to identify the mutations involved in development of resistance and its epidemiology. We expect to be able to treat MRSA infections for the foreseeable future with the help of further development of innovative compounds like PENC, identification of additional therapeutic targets, greater stewardship, and more informed decisions about combination therapy.

## Declaration of Competing Interest

The authors declare that they have no known competing financial interests or personal relationships that could have appeared to influence the work reported in this paper.
